# Ethnobotanical survey of medicinal plants used by various ethnic tribes of Mizoram, India

**DOI:** 10.1371/journal.pone.0302792

**Published:** 2024-05-10

**Authors:** Laldinfeli Ralte, Y. Tunginba Singh

**Affiliations:** Department of Botany, Laboratory of Molecular Ecology & Genetics, Mizoram University, Aizawl, Mizoram, India; Makerere University, UGANDA

## Abstract

Mizoram has a diverse range of ethnic and cultural groups, including the Lushai, Mara, Lai, Bawm, Hmar, Chakma, and Bru. Through practice and experience in their protracted battles with disease and the harsh natural environment, they have amassed rich folk medicine knowledge. The preservation of indigenous knowledge, identification of bioactive compounds, and the eventual discovery of novel medicinal plant species all depend on the documentation of the traditional uses of medicinal plants. Therefore, the present study was designed to document the ethnomedicinal knowledge related to the usage of medicinal plants in Mizoram, India. An ethnobotanical study of medicinal plants was carried out in 17 villages of 5 districts in Mizoram between September 2022 and January 2023. A total of 128 informants (77 males and 51 females) were interviewed. Information was gathered through field observations, group discussions, and semistructured interviews. The data were presented using descriptive statistics. To assess the importance of medicinal plant species, quantitative indices such as the informant consensus factor (ICF), fidelity level (FL), use value (UV), and cultural index (CI) was used. In our ethnobotanical investigation, 102 medicinal plant species from 95 genera and 58 families were recorded and documented. The majority of them (90 species) were obtained from wild habitats. The majority of the documented species were trees (48), followed by herbs (23 species) and shrubs (21 species). The most common method of preparation was decoction (67 species). Diabetes had the highest ICF value (0.81), followed by cancer, liver problems, and hypertension (0.8). The fidelity level (FL) of indigenous communities in the study area was evaluated to determine the dependability and consistency of herbal drug use. Indigenous knowledge and the variety of medicinal plant species that are used are of great values. The therapeutic applications of documented plants provide fundamental information for additional studies centered on pharmacological investigations and the preservation of the most significant species.

## Introduction

Plant resources have always been an important part of human society [1]. Since ancient times, people have used medicinal plants in both urban and rural settings, as well as increasingly in developed and developing nations [2]. The biological diversity of India is well known, with a wide variety of habitats from alpine to tropical ecosystems, a high level of endemicity, and one of the eight mega-diverse hotspots in the world [3]. There are an estimated 300,000–500,000 flora species on earth, [4] of which 50,000–80,000 flowering plant species are used for medicinal purposes worldwide [5]. The World Health Organization (WHO) estimates that approximately 80% of the population worldwide relies on herbal medicine for medical needs, particularly in rural areas [6]. Due to the lack of modern healthcare facilities in developing countries, particularly India, traditional medicines offer an affordable method of primary healthcare.

Plants have been used as a source of medicine in India since time immemorial to treat various ailments, and traditional medicine has become an integral part of the culture [7]. In general, it was estimated that 6,000 species are employed in traditional and herbal medicine in India, which corresponds to around 75% of the demands of the third world of which 23% of indigenous plants were estimated to be present in India [7]. In the meantime, 3,000 plants were formally recognized for their therapeutic qualities [7]. Even though India has a diverse cultures, this traditional knowledge is difficult to access for all individuals [8] This is because raditional knowledge is typically transmitted orally and is frequently individual-specific [9]. As a result, knowledge is frequently held by elders, heads of villages, and traditional healers in a particular community or tribe.

According to the Indian Council of Medical Research (ICMR) 2020, Mizoram is the state with the highest incidence of cancer and cardiovascular diseases in India, despite having the second-lowest population. Differentiating themselves from the rest of mainland India, its endogamous and remote populace has embraced its unique culture, way of life, and food preferences. During the past 18 years, Mizoram has had an unsettling increase in cancer and cardiovascular disease incidence and death rates, which has earned the state the unpleasant nickname “cancer capital of India”. Boiling, stewing, smoking, or fermenting are the usual methods used in Mizo traditional cuisine to prepare both vegetables and non-vegetable dishes. In addition to vegetables grown in gardens or other developed spaces, wild edible vegetables are a valuable food source. Different sections of the vegetables are used in different recipes. Although a significant proportion of rural residents are aware of the harmful consequences associated with carcinogenic substances such as smoking, eating smoked food, and drinking alcohol, problems still exist in some rural areas where access to refrigerators and other food storage solutions is restricted due to resource shortages. Therefore, it is essential to rely on fermentation and smoking as preservation techniques, with these processed foods being utilized as additions, flavors, or eaten on their own. Smoking and fermentation are two well-known methods of food preparation that are frequently used. However, ingesting such fermented and smoked foods frequently might have a major negative influence on health. In particular, a high rate of consumption has been noted, especially in the northeastern regions of India, including Mizoram. Higher incidences of stomach and lung cancer are found in Mizoram’s remote areas, where access to transportation is restricted and liquefied petroleum gas (LPG) is either extremely expensive or scarce. Using firewood for cooking is another way to address the resource shortage, however, doing so releases polycyclic aromatic hydrocarbons (PAH). The vast majority of people living in rural areas cook with wood. Health risks are exacerbated by inadequate kitchen ventilation. Unfortunately, insufficient exhaust systems plague Mizoram’s rural homes, worsening the risks associated with inadequate ventilation. For more than a century, Jhum cultivation—also known as "Shifting Cultivation"—has been practiced in Mizoram. Using this method, the land must be cleared by controlled burning, abandoned to allow for regrowth, and then repositioned.

The study area, Mizoram, India, has rich cultural and ethnic groups that include Lushai, Mara, Lai, Bawm, Hmar, Chakma, and Bru. In Mizoram, 302 medicinal plants were recorded as being used by the indigenous people [10]. The people from these various ethnic groups use plants for their primary health care, traditional ceremonies, industrial materials, food sources, and building materials in most of the villages. The southern part of Mizoram has been inhabited by various ethnic groups, most of the villages are still underdeveloped without proper power supply, and a lack of primary health centers is still very common. Most of the study areas were remote places where no modern healthcare facilities were available, which forced them to rely mainly on traditional herbal medicines. However, agricultural development, deforestation, inadequate recording, and oral transmission of traditional knowledge pose threats to indigenous knowledge and medicinal plants among ethnic groups. Some works had been attempted earlier on the ethnomedicinal plants of Mizoram, where the qualitative data were highlighted mainly with particular ethnic groups (Lushai, and Chakma) [11–14]. Apart from the studied ethnic groups, there are still some tribes such as Chawrei, Darlong, Paite, Thado, and Chin tribes that are understudied. However, there are no reports regarding the ethnobotanical aspect of medicinal plants used among various ethnic groups with in-depth studies in Mizoram. Therefore, in the present study, an attempt was made to document the ethnomedicinal knowledge along with medicinal plant species used by various ethnic groups. Further, quantitative analysis, and comparison of therapeutic plants knowledge among various ethnic groups, threat to plant resources and conservation status of medicinal plants in the Mizoram were also studied.

## Materials and methods

### Study area

**Fig 1:**
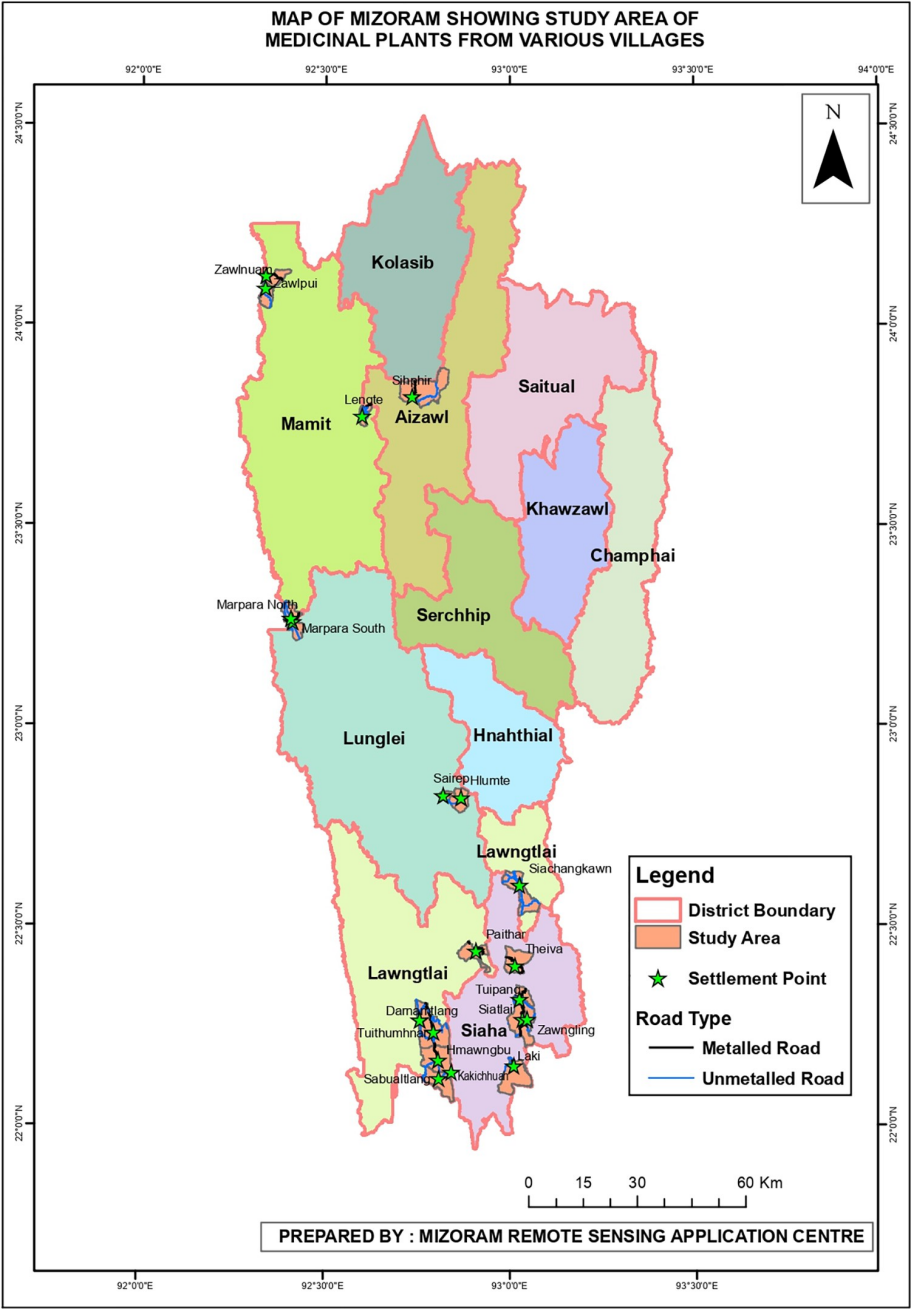
Map of Mizoram showing the present study area (Photo courtesy: Mizoram Remote Sensing Application Centre).

Mizoram shares two international borders with Bangladesh in the west and Myanmar in the east and is located in the Indo-Burma biodiversity hotspot region. The study was carried out in various villages of the Aizawl, Mamit, Lunglei, Lawngtlai, and Saiha districts, and the ethnic groups in the locality included Lushai, Bru, Chakma, Lai, Bawm, and Mara (Fig 1). Since there has not been any prior research done on the ethnomedicinal plants and quantitative indices among different ethnic groups from these areas, the current study was carried out the present work from these study areas. The study area lies between latitude 23.1645°N and longitude 92.9376°E with an annual rainfall of 2500–3000 mm. The present study was conducted from September 2022 –January 2023. Most of the inhabitants in the study area rely on agriculture for their livelihood. According to altitude and rainfall, Champion and Seth [15] described the vegetation types of the Mizoram into three categories: tropical wet-evergreen forest, montane subtropical forest, and temperate forest. The study areas were categorized as remote areas with limited access to healthcare, which force the residents to rely on alternative treatments. The fact that traditional healers still rely heavily on herbal remedies to treat a variety of ailments shows the importance/relevance of traditional medicines compared to modern healthcare systems. It is believed that more effort needs to be put into prospecting for and researching the medicinal plants that are crucial to the Mizo tribes’ ability to maintain good health.

### Informant selection and data collection

**Fig 2:**
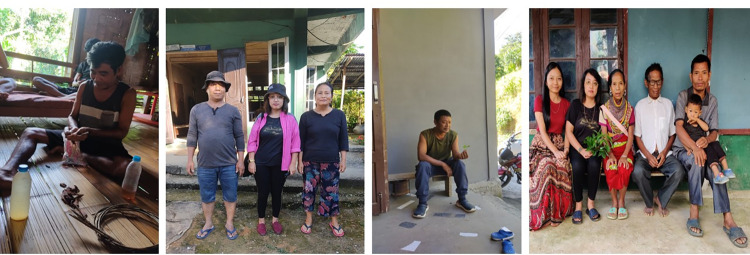
Informants from the study area with the author.

**Fig 3:**
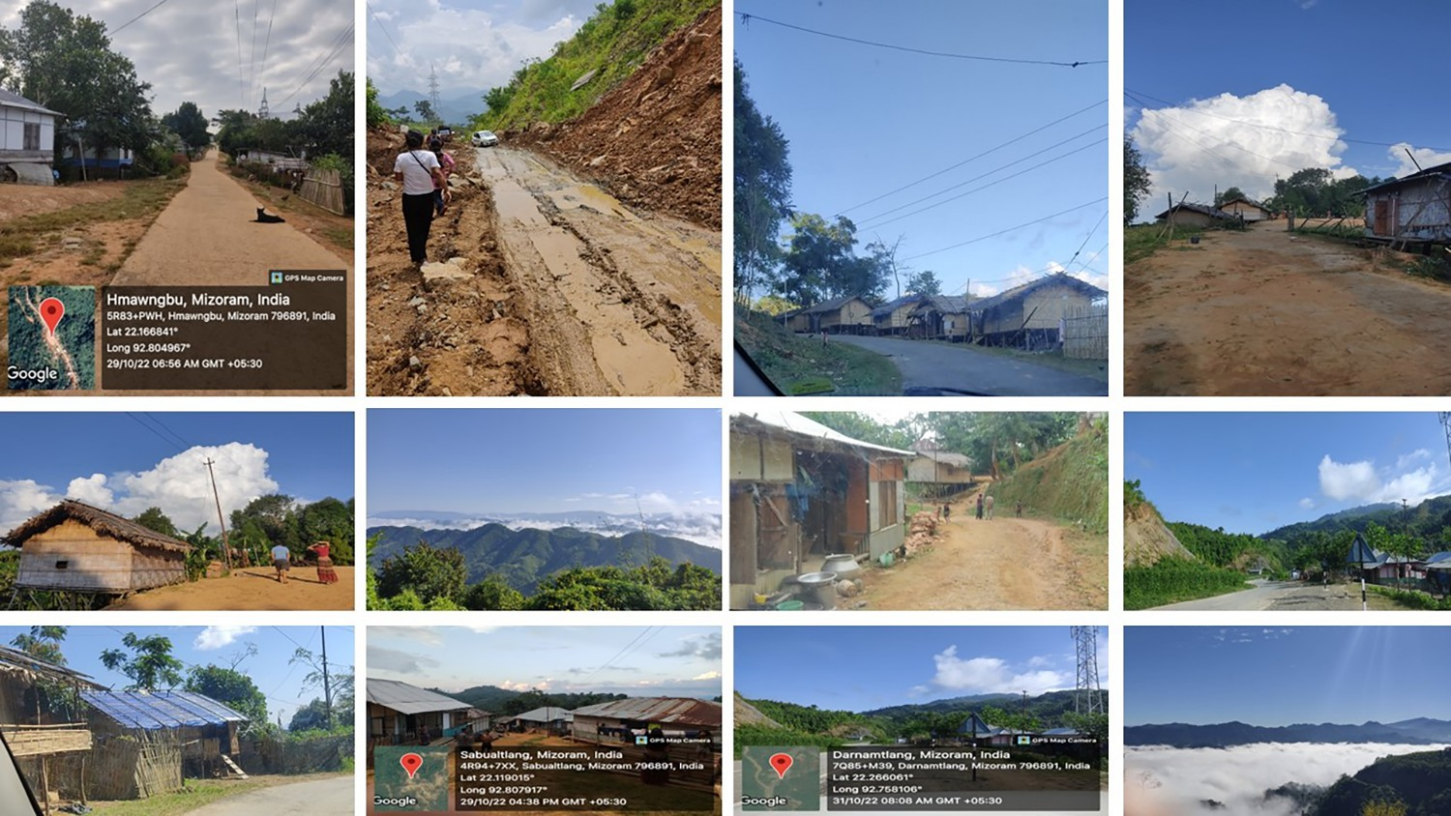
Selected pictures of the study sites from different villages inhabitant by various ethnic groups.

Before collecting the data, a reconnaissance survey was conducted to acquire information from the studied district’s administration office, and other people in the study region. This information was used to choose the study sites and informants. Ethnobotanical study was carried out in 17 villages i.e., Sihphir (23.8179°N, 92.7369°E), Zawlnuam (24.1351°N, 92.3345°E), Zawlpui (24.1199°N, 92.23341°E), Sairep (22.8229°N, 92.8211°E), Hlumte (22.8195°N, 92.8679°E), Siachangkawn (22.815989°N, 93.0263°E), Paithar (22.5333°N, 92.8999°E), Hmawngbu (22.1622°N, 92.8066°E), Tuithumhnar (22.2310°N, 92.7917°E), Kakichhuah (28.6689°N, 77.2112°E), Sabualtlang (22.3762°N, 92.7508°E), Darnamtlang (22.2814°N, 92.7537°E), Tuipang (22.3146°N, 93.0251°E), Theiva (22.5198°N, 93.0441°E), Zawngling (22.2737°N, 93.0331°E), Laki (22.1527°N, 93.0088°E), and Siatlai (22.2653°N, 93.0338°E) selected purposively from a total of 5 districts in the study based on the recommendation of the local elders, authorities, the occurrence of ethnic groups, and availability of traditional healers. A total of 128 informants (77 males and 51 females) were interviewed in the study area (Fig 2) from 17 villages (Fig 3) (i.e., Sihphir– 3; Zawlnuam– 8; Zawlpui– 5; Sairep– 9; Hlumte– 7; Siachangkawn– 5; Paithar– 7; Hmawngbu– 11; Tuithumhnar– 7; Kakichhuah– 7; Sabualtlang– 6; Darnamtlang– 7; Tuipang– 7; Theiva– 7; Zawngling– 1-; Laki– 15; Siatlai– 7) due to the presence of local healers, size of populations, and occurrence of ethnic groups. Informants were selected with purposive, snowball and random sampling methods following previous publication [4]. A 113 representative common participants and 15 knowledgeable traditional healers (key participants) from the study areas were chosen using random and purposeful sampling techniques, respectively [16]. The traditional experts who are the keepers of local knowledge on medicinal plants, such as local healers, automatically qualify as key participants. All the participants received an explanation of the process and nature of this study and were asked to provide oral informed consent.

An ethnobotanical approach was used to explore the traditional practitioner’s knowledge, diagnosis, and treatment practices for various ailments. In this section of the study, data were gathered using semistructured and structured interview protocols following the established protocol [17]. Through semistructured interviews, field visits, observations, and group discussions in the study area, information about the medicinal uses of plants was gathered. During the interviews with the informants, the local names of the plants, the ailments treated by the plants, the plant parts used, the condition of the plant material, the modes of preparation, and the routes of administration were meticulously recorded and adopted following Martin [16]. A set of prepared questionnaires approved by the Ethics Review Committee served as the basis for the interviews. Before undertaking the ethnobotanical survey, informed consent was sought from the heads of each village, and specific informants. Field studies and interviews were then conducted following the International Ethnobiology Society’s ethical standard (ISE Code of Ethics 2008). Additional data were also gathered, including the name, age, profession, and educational background of the informants. The preservation of medicinal plants and traditional knowledge as well as their threats were the topics of group discussions. The present study will form an input for strengthening knowledge on medicinal plants that are being utilized by the indigenous people, for medicinal plant protection and for further phytochemical investigation for new drug formulations.

### Identification and specimen collection

Two branches/plants were collected for voucher specimens. The authors got permission from the land owner, and forest officers from the reserve and protected areas. The morphological characteristics and habitats of each medicinal plant species were documented through field observations with traditional healers. Local medicinal plants were gathered as voucher specimens and photographed, and their habitats and habits were documented. The specimens were identified using Flora of Mizoram [13] and botanical websites such as the plant list (www.theplantlist.org/accessed 6-7/02/2023) and the world flora (www.worldfloraonline.org/ accessed 6-7/02/2023) and confirmed by taxonomic experts from the Department of Botany, Mizoram University. The plant specimens (with their local name, collection number, collection date, name of collector, location and plant description) were pressed, dried, and labelled and then deposited in the Herbarium of the Department of Botany, Mizoram University for future reference.

### Data analysis

We utilized the ethnobotanyR package [18] to carry out quantitative analysis of the obtained data. Various quantitative calculations were performed in particular to assess the importance of ethnomedicinal plants for the investigated study area. We quantified informant consensus factor (ICF) [19,20], fidelity level (FL) [21], use value (UV) [22], and cultural value (CV) [22]. This ethnobotanical documentation included 10 different use or disease categories that were developed and modified from the ICD-11 (International Classification of Diseases) for Mortality and Morbidity statistics proposed by WHO (2022). These estimates combined to provide a thorough assessment of the significance of plants for the investigated local communities.

Statistical analysis and graph plotting was done using GraphPad Prism ver 8.0, the Microsoft Excel spreadsheet 2019, and R-4.2.2.2 software (R Development Core Team; Venice, Italy) using various CRAN packages [23]. To determine the number of medicinal plant species and related uses reported by key and general informants, an independent sample *t*-test was performed. The indigenous knowledge disparity between males and females on the number of medicinal plant species and associated uses they stated was also identified using this method. A t-test using an independent sample was used to calculate the variation in the reported numbers of medicinal plant species and their associated uses between the two age groups. Further, a Venn diagram was created using Jvenn software (https://jvenn.toulouse.inrae.fr/app/index.html) to determine cross-cultural significance.

## Results

### Socio-demographic characteristics of respondents

In the present study, the indigenous knowledge of medicinal plants utilized by various ethnic groups such as Lushai, Mara, Lai, Bawm, Hmar, Chakma, and Bru was documented. Among these groups, 113 general informants (Lushai-38; Mara-29; Lai-25; Bawm-10; Hmar-6; Chakma-15; Bru-5), and 15 key informants (Lushai-3; Mara-2; Lai-2; Bawm-2; Hmar-2; Chakma-2; Bru-2) were selected depending upon the abundance of ethnic groups from the study area. The age, gender, profession, and educational level of informants from the study area are shown in Table 1. The age of the informants ranged from 21–84, where 37.5% of the informants were in the age range of 51–70, 28.91% had a high school level education, and 43.75% were self-employed (farmers, business owners, etc.). Among the informants, 11.71% were key informants while 88.28% were general informants. The majority of the informant stated that they acquired most of their information covertly from relatives that came from their ancestors. There was no statistically significant difference in knowledge (*p*>0.05) between male and female informants, profession, and educational level in the study region when it came to medicinal plant knowledge, according to a two-tailed independent sample t-test (Table 1). However, there was a significant difference (*p*<0.05) between key and general informants in terms of the average number of medicinal plants utilized and the knowledge in the study area. The key informants had greater knowledge of medicinal plants than the general informants (Table 1). Further, there was also a significant difference in age-related medicinal plant knowledge in the study area.

**Table 1:** Demographic features of informants.

Factor	Categories	Number	Frequency (%)	Mean	*P* value
Gender	Male	77	60.2%	5.6	0.98
	Female	51	39.8	5.3	
Profession	Government employed	42	32.8	2.3	0.86
	Self-employed	56	43.8	2.2	
	Unemployed	30	23.4	2.9	
Age	<30	24	18.8	1.9	0.005*
	31–50	33	25.8	2.9	
	51–70	48	37.5	2.8	
	>71	23	17.9	4.4	
Educational level	Primary	16	12.5	6.4	0.96
	Middle	25	19.5	5.1	
	High School	37	28.9	5.7	
	Higher Secondary	22	17.2	4.9	
	Graduate & above	28	21.9	6.8	
Informant category	Key informant	15	11.7	11.4	0.002*
	General informant	113	88.2	6.7	

Where

* denotes significance level p>0.05.

### Diversity of medicinal plants, their habitats, and growth form

A total of 102 medicinal plant species belonging to 95 genera and 58 families were identified and documented from the study areas. The ethnomedicinal uses of each species, botanical name, local name, family, habit, habitat, parts used, and method of use were also recorded (Table 2).

**Table 2:** List of medicinal plants documented from the study areas.

Plant species number	Botanical Name	Local name	Family	Habit	Habitat	Part used	Ailment	Method of preparation and administration	Use Value (UV)
**1.**	*Barleria cristata* L. (MZU/BOT/218)	Par pawl (Lushai)	Acanthaceae	Herb	Wild	Whole plant	Diabetes	Grounded; oral	0.1
**2.**	*Phlogacanthus thyrsiformis* (Roxb.ex Hardw.) Mabb. (MZU/BOT/283)	Khumtiangkohha (Lushai)	Acanthaceae	Shrub	Wild	Root, leaves	Ulcer, cancer	Decoction; oral	1
**3.**	*Celosia argentea* L. (MZU/BOT/228)	Zamzo (Lushai); Ranga chuma (Chakma)	Amaranthaceae	Shrub	Home garden	Flower, leaves	haemorrhage, dysentery, cancer	Paste, Decoction; oral; external applied	0.3
**4.**	*Achyranthes bidentata* Blume (MZU/BOT/204)	Vangvat hlo (Lushai)	Amaranthaceae	Shrub	Wild	Leaves	Stomach cancer	Decoction; oral	0.4
**5.**	*Lannea coromandelica* (Houtt.) Merr. (MZU/BOT/266)	Tawitawsuak (Lushai)	Anacardiaceae	Tree	Wild	Bark, leaves	Chronic ulcer, wounds	Ointment; external applied	0.2
**6.**	*Dasymaschalon longiflorum* Finet & Gagnep. (MZU/BOT/236)	Chiripi (Bru)	Annonaceae	Tree	Wild	Root	Dysentery, Chronic ulcer	Grounded; oral	0.2
**7.**	*Alstonia scholaris* (L.) R. Br. (MZU/BOT/211)	Thuamriat (Lushai)	Apocynaceae	Tree	Wild	Bark	Dysentery, Stomach ulcer, Hypertension	Decoction; oral	0.3
**8.**	*Rauvolfia serpentina* Benth. ex Kurz (MZU/BOT/290)	Thingzungkha (Lushai)	Apocynaceae	Shrub	Wild	Root	Stomachache, hypertension	Decoction; oral	0.1
**9.**	*Ruehssia macrophylla* (Humb. & Bonpl. ex Schult.) H. Karst. (MZU/BOT/292)	Ankhapui (Lushai, Hmar)	Apocynaceae	Tree	Wild	Leaves, stem	Stomachache, hypertension	Decoction; oral	0.6
**10.**	*Amorphophallus paeoniifolius* (Dennst.) Nicolson (MZU/BOT/213)	Ba telhawng (Lushai)	Araceae	Shrub	Wild	Corm/rhizome	Tumor, constipation	Decoction; oral	0.1
**11.**	*Trevesia palmata* Vis. (MZU/BOT/297)	Kawhtebel (Lushai, Hmar)	Araliaceae	Tree	Cultivated	Fruit, leaf stalk	Stomachache, hypertension	Decoction; oral	0.3
**12.**	*Arenga pinnata* Merr. (MZU/BOT/217)	Thangtung(Lushai)	Arecaceae	Tree	Wild	Root	Bronchitis, Stomachache	Powder form; oral	0.1
**13.**	*Dracaena spicata* Roxb. (MZU/BOT/237)	Phunhring (Lushai)	Asparagaceae	Shrub	Wild	Root	Stomachache	Grounded, eaten as raw; oral	0.1
**14.**	*Polygonatum cirrhifolium* (Wall.) Royle (MZU/BOT/288)	Hnah kawi (Lushai)	Asparagaceae	Herb	Wild	Root	Ulcer, bronchitis	Decoction; oral;	0.2
**15.**	*Artemisia vulgaris* L. (MZU/BOT/216)	Sai (Lushai, Hmar, Mara)	Asteraceae	Herb	Wild	Root	Fever, Stomachache	Grounded; oral	0.4
**16.**	*Blumea lanceolaria* Druce (MZU/BOT/221)	Buarze (Lushai)	Asteraceae	Herb	Home garden	Leaves	Cancer, chronic ulcer, dysentery	Grounded/crushed, infusion; oral	0.5
**17.**	*Blumea sinuata* (Lour.) Merr. (MZU/BOT/222)	Khuanglawr (Lushai)	Asteraceae	Herb	Wild	Leaves	Heart disease	Decoction; oral	0.2
**18.**	*Cirsium shansiense* Petr. (MZU/BOT/230)	Lenhling (Lushai)	Asteraceae	Herb	Wild	Roots	wounds, haemorrhage, stomach ulcer	Paste; external applied, oral	0.2
**19.**	*Enydra fluctuans* Lour. (MZU/BOT/245)	Par hring (Hmar)	Asteraceae	Herb	Wild	Whole plant	Hypertension	Crushed; oral	0.2
**20.**	*Laggera crispata* (Vahl) Hepper & J.R.I.Wood (MZU/BOT/265)	Ramvaihlo (Lushai)	Asteraceae	Herb	Wild	Leaves	Chronic ulcer	Ointment; external applied	0.2
**21.**	*Berberis napaulensis* (DC.) Laferr. (MZU/BOT/220)	Pualleng(Lushai, Mara)	Berberidaceae	Shrub	Wild	Bark	Stomachache	Decoction; oral	0.2
**22.**	*Podophyllum hexandrum* Royle (MZU/BOT/287)	Par var (Lushai)	Berberidaceae	Shrub	Wild	Rhizome	Cancer	Decoction; oral	0.3
**23.**	*Stereospermum fimbriatum* DC. (MZU/BOT/294)	Zihaw (Hmar, Mara)/zihnghal (Lushai)	Bignoniaceae	Tree	Wild	Shoot	Stomachache, chronic ulcer	Decoction; oral	0.2
**24.**	*Cordia dichotoma* G. Forst. (MZU/BOT/233)	Muk (Lushai, Hamr)	Boraginaceae	Tree	Wild	Bark, Leaves	Stomachache, ulcer	Decoction; oral	0.1
**25.**	*Heliotropium indicum* L. (MZU/BOT/261)	Ni zawn par (Lushai, Hmar)	Boraginaceae	Herb	Wild	Leaves	Chronic ulcer	Decoction; oral	0.2
**26.**	*Canna indica* L. (MZU/BOT/224)	Kungpuimuthi (Lushai)	Cannaceae	Herb	Wild	Flower	Diabetes	Decoction; oral	0.2
**27.**	*Lobelia angulata* G. Forst (MZU/BOT/273)	Choakthi (Lushai)	Campanulaceae	Herb	Wild	Whole plant	Stomach ulcer, hypertension when boiled with *Centella asicatica* (L.) Urb.	Decoction; oral	0.4
**28.**	*Lonicera macrantha* Spreng. (MZU/BOT/274)	Leihruisen (Lushai)	Caprifoliaceae	Shrub	Wild	Leaves,roots, whole plant	Stomach ulcer, hypertension, oesophagus cancer, bone cancer	Decoction, ointment when crush with *Mikania micrantha* (L.) Willd., and *Chromolaena odorata* (L.) R.M.King & H.Rob.; oral, extermal applied	0.5
**29.**	*Getonia floribunda* Roxb. (MZU/BOT/253)	Hruisen (Hmar, Lushai)	Combretaceae	Tree	Wild	Roots	Stomach ulcer	Grounded; external applied	0.3
**30.**	*Terminalia phillyreifolia* (Van Heurck & Mull. Arg.) Gere & Boatwr. (MZU/BOT/295)	Zairum (Lushai, Hmar)	Combretaceae	Tree	Wild	Bark	Diarrhoea, Stomach ulcer	Decoction; oral	0.4
**31.**	*Garcinia lanceifolia* Roxb. (MZU/BOT/251)	Pelhte (Lushai)	Clusiaceae	Tree	Wild	Leaves	Stomachache	Infusion; oral	0.5
**32.**	*Benincasa hispida* Cogn. (MZU/BOT/219)	Maipawl (Lushai, Hmar, Mara)	Cucurbitaceae	Climbing shrub	Cultivated	Fruit	Diabetes	Decoction; oral	0.4
**33.**	*Trichosanthes cucumerina* L. (MZU/BOT/298)	Berul Lushai, Lai)	Cucurbitaceae	Climbing shrub	Cultivated	Fruit	Cancer	Decoction; oral	0.3
**34.**	*Zanonia indica* L. (MZU/BOT/300)	Lalruanga dawibur (Lushai)	Cucurbitaceae	Climbing shrub	Wild	Fruit	Stomachache, ulcers, asthma	Infusion; oral	0.5
**35.**	*Dillenia indica* L. (MZU/BOT/238)	Kawrthindeng (Lushai)	Dilleniaceae	Tree	Wild	Bark, Leaves, Fruit	Fever, diarrhoea, cancer	Grounded, decoction; oral	0.3
**36.**	*Dillenia pentagyna* Roxb. (MZU/BOT/239)	Kaihzawl (Lushai)	Dilleniaceae	Tree	Wild	Bark	Stomach ulcer	Decoction; oral	0.2
**37.**	*Tetracera sarmentosa* Vahl (MZU/BOT/296)	Hruithindeng (Lushai)	Dilleniaceae	Tree	Wild	Bark	Stomachache	Decoction; oral	0.3
**38.**	*Dipterocarpus turbinatus* C.F. Gaertn. (MZU/BOT/242)	Lawngthing (Lushai)	Dipterocarpaceae	Tree	Wild	Bark	Chronic ulcer	Decoction; oral	0.2
**39.**	*Diospyros variegata* Kurz (MZU/BOT/241)	Thinghang (Lai)	Ebenaceae	Tree	Wild	Root, bark	Ulcer, diarrhoea, dysentery	Decoction; oral	0.2
**40.**	*Claoxylon khasianum* Hook.f. (MZU/BOT/232)	Nagabang (Lushai)	Euphorbiaceae	Shrub	Wild	Root	Tumour, cancer	Paste; external applied	1
**41.**	*Mallotus leucocarpus* (Kurz) Airy Shaw (MZU/BOT/277)	Sikiah (Lushai)	Euphorbiaceae	Tree	Wild	Root	Stomachache	Decoction; oral	0.2
**42.**	*Mallotus philippensis* (Lam.) Mull.Arg. (MZU/BOT/278)	Thingkhei (Lushai)	Euphorbiaceae	Tree	Wild	Bark, root	Diarrhoea, stomachache	Decoction; oral	0.1
**43.**	*Mallotus roxburghianus* Mull.Arg. (MZU/BOT/279)	Zawngtenawhlung (Lushai)	Euphorbiaceae	Shrub	Wild	Leaves, root	Diabetes, hypertension	Decoction; oral	0.1
**44.**	*Aganope thyrsiflora* (Benth.) Polhill (MZU/BOT/208)	Hulhu (Hmar, Lushai)	Fabaceae	Climbing shrub	Wild	Fruit	Dysentery, Stomach ulcer	Decoction; oral	0.2
**45.**	*Albizia odoratissima* (L.f.) Benth. (MZU/BOT/209)	Kangtek (Lushai)	Fabaceae	Tree	Wild	Bark	Ulcers, leprosy	Crushed; external applied	0.2
**46.**	*Entada rheedei* Spreng. (MZU/BOT/244)	Kawi (Lushai, Hmar, Bawm)	Fabaceae	Tree	Wild	Bark	Chronic ulcer	Grounded; external applied	0.3
**47.**	*Erythrina stricta* Roxb. (MZU/BOT/246)	Fartuah (Lushai)	Fabaceae	Tree	Wild	Bark	Chronic ulcer, cancer	Decoction; oral	0.4
**48.**	*Lasiobema scandens* (L.) de Wit (MZU/BOT/268)	Zawngaleihlawm (Lushai)	Fabaceae	Shrub	Wild	Leaf	Stomachache	Decoction; oral	0.1
**49.**	*Pongamia pinnata* (L.) Merr. (MZU/BOT/289)	Tel thing (Lushai)	Fabaceae	Shrub	Wild	Whole plant	Ulcer, diarrhoea, diabetes, leprosy	Decoction; oral	0.2
**50.**	*Mucuna pruriens* (L.) DC. (MZU/BOT/281)	Uitema (Bawm)	Fabaceae	Climbing shrub	Wild	Leaves, fruit	Kidney problem, stomach ulcer, liver problem	Infusion; oral	0.2
**51.**	*Xylia xylocarpa* (Roxb.) W. Theob. (MZU/BOT/299)	Thinguk (Lushai)	Fabaceae	Tree	Wild	Bark	Diarrhoea, Stomach ulcer	Decoction; oral	0.2
**52.**	*Castanopsis echinocarpa* Miq. (MZU/BOT/226)	Thingsia (Lushai)	Fagaceae	Tree	Wild	Bark	Stomach ulcer	Decoction; oral	0.2
**53.**	*Aeschynanthus parviflorus* Spreng. (MZU/BOT/207)	Bawltehlantai (Lushai)	Gesneriaceae	Epiphyte	Wild	Root, leaf, seed, flower	Inflammation, stomachache, cardiac disorder, asthma, breast cancer	Grounded/crushed; oral, external applied	0.1
**54.**	*Gelsemium elegans* (Gardner & Champ.) Benth. (MZU/BOT/252)	Hnamtur (Lushai)	Gelsemiaceae	Climbing shrub	Wild	Roots	Stomach ulcer	Grounded; oral	0.4
**55.**	*Curculigo crassifolia* (Baker) Hook.f. (MZU/BOT/234)	Phaiphak (Lushai)	Hypoxidaceae	Shrub	Wild	Rhizome	Stomachache	Grounded; external applied	0.2
**56.**	*Callicarpa arborea* Roxb. (MZU/BOT/223)	Hnahkiah (Lushai)	Lamiaceae	Shrub	Wild	Bark	Haemorrhage, Stomach ulcer, Diabetes	Grounded; external applied	0.2
**57.**	*Gmelina arborea* Roxb. (MZU/BOT/255)	Thlanvawng (Lushai)	Lamiaceae	Tree	Wild	Leaves	Stomach ulcer	Grounded; external applied	0.2
**58.**	*Ocimum gratissimum* L. (MZU/BOT/282)	Khumbangbang (Mara)	Lamiaceae	Herb	Home garden	Whole plant	Diabetes	Decoction; oral	1
**59.**	*Litsea glutinosa* (Lour.) C.B.Rob (MZU/BOT/272)	Parsen (Lushai)	Lauraceae	Tree	Wild	Bark	Heart disease	Decoction; oral	0.1
**60.**	*Lagerstroemia speciosa* Pers. (MZU/BOT/264)	Thlado (Lushai)	Lythraceae	Tree	Wild	Bark	Chronic ulcer	Decoction; oral	0.2
**61.**	*Magnolia champaca* (L.) Baill.ex Peirre (MZU/BOT/276)	Ngiau (Lushai)	Magnoliaceae	Tree	Wild	Flowers, fruit	Stomachache	Infusion; oral	0.1
**62.**	*Abelmoschus moschatus* Medik. (MZU/BOT/201)	Ui chhu hlo (Lushai)	Malvaceae	Herb	Wild	Seed	Stomachache	Powder form; external applied	0.2
**63.**	*Abutilon indicum* (L.) Sweet (MZU/BOT/202)	Mai an suak (Lushai)	Malvaceae	Shrub	Wild	Leaves	Cancer, Diabetes	Decoction; oral	0.4
**64.**	*Ceiba pentandra* (L.) Gaertn. (MZU/BOT/227)	Japanpang (Lushai)	Malvaceae	Tree	Wild	Fruit	Diabetes	Decoction; oral	0.2
**65.**	*Chukrasia tabularis* A. Juss. (MZU/BOT/229)	Zawngtei (Lushai)	Meliaceae	Tree	Wild	Bark, Fruit	Dysentery, diarrhoea, fever, stomachache	Juice, grounded; oral, external applied	0.3
**66.**	*Dysoxylum excelsum* Blume (MZU/BOT/243)	Thingthupui (Lushai, Hmar)	Meliaceae	Tree	Cultivated	Leaves, stem	Stomachache	Decoction; oral	0.5
**67.**	*Ficus racemosa* L. (MZU/BOT/248)	Thei chek (Lushai)	Moraceae	Tree	Wild	Root, bark, fruit	dysentery, diabetes, diarrhoea, stomachache	Decoction, grounded, eaten as raw; oral	0.2
**68.**	*Ficus semicordata* Buch.-Ham.ex Sm. (MZU/BOT/249)	Thenpui (Lushai)	Moraceae	Tree	Wild	Bark	Liver ailment	Decoction; oral	0.2
**69.**	*Mirabilis jalapa* L. (MZU/BOT/280)	Artukkhuan (Lushai)	Nyctaginaceae	Shrub	Home garden	Root	Diabetes	Decoction; oral	0.2
**70.**	*Jasminum nervosum* Lour. (MZU/BOT/262)	Hrurkha (Lushai)	Oleaceae	Shrub	Wild	Leaves	Stomachache, fever	Decoction; oral	0.2
**71.**	*Lepionurus sylvestris* Blume (MZU/BOT/271)	Anpangthuam (Lushai)	Opiliaceae	Shrub	Wild	Leaf	Diabetes, Cancer, hypertension	Decoction; oral	0.2
**72.**	*Argemone mexicana* L. (MZU/BOT/215)	Berbek (Lushai)	Papaveraceae	Shrub	Wild	Leaves	Cancer, tumor, inflammation	Decoction; oral	0.1
**73.**	*Eurya acuminata* DC. (MZU/BOT/247)	Sihneh (Lushai)	Pentaphylaceae	Tree	Wild	Leaves	Stomchache	Decoction; oral	0.5
**74.**	*Piper attenuatum* Buch.-Ham.ex Wall. (MZU/BOT/285)	Pawhrual (Lushai)	Piperaceae	Climbing shrub	Wild	Leaves	Stomachache	Infusion; oral	0.2
**75.**	*Plantago major* L. (MZU/BOT/286)	Kelbaan (Lushai)	Plantaginaceae	Shrub	Wild	Leaves	Chronic ulcer, diabetes	Decoction; oral	0.6
**76.**	*Maesa montana* A.DC. (MZU/BOT/275)	Arngeng (Lushai)	Primulaceae	Tree	Wild	Shoot	Dysentery, Stomachache	Decoction; oral	0.1
**77.**	*Helicia excelsa* Blume (MZU/BOT/258)	Sialhma (Lushai)	Proteaceae	Tree	Wild	Bark	Stomachache	Decoction; oral	0.3
**78.**	*Helicia robust*a (Roxb.) Blume (MZU/BOT/259)	Pasaltakaza (Lushai)	Proteaceae	Tree	Wild	Root	Stomach ulcer, diabetes	Decoction; oral	0.3
**79.**	*Adiantum philippense* L. (MZU/BOT/206)	Lungpuisam (Lushai)	Pteridaceae	Herb	Wild	Leaves	Dysentery, Fever, Stomach ulcer, Diabetes	Decoction; oral	0.1
**80.**	*Aporosa octandra* (Buch.-Ham. ex D. Don) Vickery (MZU/BOT/214)	Chhawntual (Lushai)	Phyllanthaceae	Shrub	Wild	Bark	Stomach ulcer	Decoction; oral	0.1
**81.**	*Flueggea virosa* (Roxb. ex Willd.) Royle (MZU/BOT/250)	Saisiak (Lushai)	Phyllanthaceae	Tree	Wild	Leaves	Diabetes	Grounded, external applied	0.1
**82.**	*Phyllanthus fraternus* G.L.Webster (MZU/BOT/284)	Mitthisunhlu (Lushai)	Phyllanthaceae	Herb	Wild	Whole plant	Diabetes, hepatitis	Decoction; oral	0.5
**83.**	*Aconitum heterophyllum* Wall. (MZU/BOT/205)	Sanghar tur (Lushai)	Ranunculaceae	Herb	Wild	Root	Diarrhoea, stomachache, diabetes	Decoction; oral	0.1
**84.**	*Ziziphus mauritiana* Lam. (MZU/BOT/302)	Bawrai (Lushai, Hmar)	Rhamnaceae	Tree	Home garden	Root	Chronic ulcer	Decoction; oral	0.1
**85.**	*Laurocerasus undulata* (Buch.-Ham.ex D.Don) M. Roem. (MZU/BOT/269)	Theiarlung (Lushai)	Rosaceae	Tree	Wild	Bark	Heart disease	Decoction; oral	0.2
**86.**	*Hedyotis scandens* Roxb. (MZU/BOT/257)	Kelhnamtur (Lushai)	Rubiaceae	Herb climber	Wild	Leaves	Kidney problem, stomachache	Decoction, grounded, eaten as raw; oral	0.4
**87.**	*Rubia cordifolia* L. (MZU/BOT/291)	Rawng thlak (Lushai)	Rubiaceae	Herb	Wild	Root, stem	Dysentery, ulcer, inflammation	Decoction; oral	0.1
**88.**	*Citrus aurantium* L. (MZU/BOT/231)	Sisu (Lushai)	Rutaceae	Tree	Home garden	Fruit	Stomchache	Juice; oral	0.2
**89.**	*Zanthoxylum armatum* DC. (MZU/BOT/301)	Ar hrik reh (Lushai)	Rutaceae	Tree	Wild	Bark, fruit	Tumour,diarrhoea, fever	Decoction; oral	0.2
**90.**	*Acer oblongum* Wall. Ex DC. (MZU/BOT/203)	Thingphingphihlip (Lushai)	Sapindaceae	Shrub	Wild	Bark	Stomachache	Decoction; oral	0.1
**91.**	*Dimocarpus longan* Lour. (MZU/BOT/240)	Theifeimung (Lushai)	Sapindaceae	Tree	Cultivated	Fruit	Fever, Stomachache	Eaten as raw; oral	0.3
**92.**	*Smilax glabra* Roxb. (MZU/BOT/293)	Tluangngil (Lushai)	Smilacaceae	Shrub	Wild	Rhizome	Stomachache	Decoction; oral	0.2
**93.**	*Girardinia diversifolia* (Link) Friis (MZU/BOT/254)	Kangthai (Lushai)	Urticaceae	Shrub	Wild	Root	Chronic ulcer, diabetes	Decoction; oral	0.2
**94.**	*Lantana camara* L. (MZU/BOT/267)	Hlingpangpar (Lushai)	Verbenaceae	Herb	Wild	Leaves	wounds, ulcers, cuts	Ointment; external applied	0.1
**95.**	*Leea compactiflora* Kurz (MZU/BOT/270)	Kawlkar (Lushai)	Vitaceae	Shrub	Wild	Bark	Stomachache	Decoction; oral	1
**96.**	*Alpinia calcarata* (Haw.) Roscoe (MZU/BOT/210)	Aichal (Lushai)	Zingiberaceae	Shrub	Wild	Rhizome	Stomach ulcer	Decoction; oral	0.2
**97.**	*Amomum maximum* Roxb. (MZU/BOT/212)	Aidu (Lushai)	Zingiberaceae	Herb	Wild	Rhizome	Hypertension	Decoction; oral	0.4
**98.**	*Cautleya gracilis* (Sm.) Dandy (MZU/BOT/225)	Pa le (Lushai)	Zingiberaceae	Herb	Wild	Rhizome	Stomachache	Eaten as raw; oral	0.2
**99.**	*Curcuma caesia* Roxb. (MZU/BOT/235)	Ailaidum (Lushai)	Zingiberaceae	Herb	Wild	Rhizome	Stomachache, diarrhoea, dysentery	Grounded; external applied, oral	0.2
**100.**	*Hedychium spicatum* Buch.-Ham. ex Sm. (MZU/BOT/256)	Aithur (Lushai)	Zingiberaceae	Herb	Wild	Rhizome	liver ailment, diarrhoea, stomachache	Grounded; external applied	0.3
**101.**	*Hellenia speciosa* (J. Koenig) Govaerts (MZU/BOT/260)	Sumbul (Lushai)	Zingiberaceae	Herb	Wild	Rhizome	Kidney problem, stomachache	Decoction; oral	0.4
**102.**	*Kaempferia rotunda* Don (MZU/BOT/263)	Tuktinpar (Lushai)	Zingiberaceae	Herb	Wild	Root	Stomachache	Grounded; oral	0.2

Among the identified plants, Fabaceae had the highest number of plant species (8), followed by Zingiberaceae (7), Asteraceae (6), and Euphorbiaceae (4). Three (3) species each were found from the families Malvaceae, Apocynaceae, Phyllanthaceae, Lamiaceae, and Dilleniaceae, while Sapindaceae, Amaranthaceae, Acanthaceae, Berberidaceae, Meliaceae, Rutaceae, Boraginaceae, Asparagaceae, Moraceae, Combretaceae, Rubiaceae, and Proteaceae had 2 species each, and the remaining 37 families had one species each.

The local people gather medicinal plants from various places such as wild, home gardens, and cultivated lands. In the present study, out of 102 therapeutic plants 89.2% were collected from the wild, 4.9% from home gardens, and 5.8% from cultivated lands.

**Fig 4:**
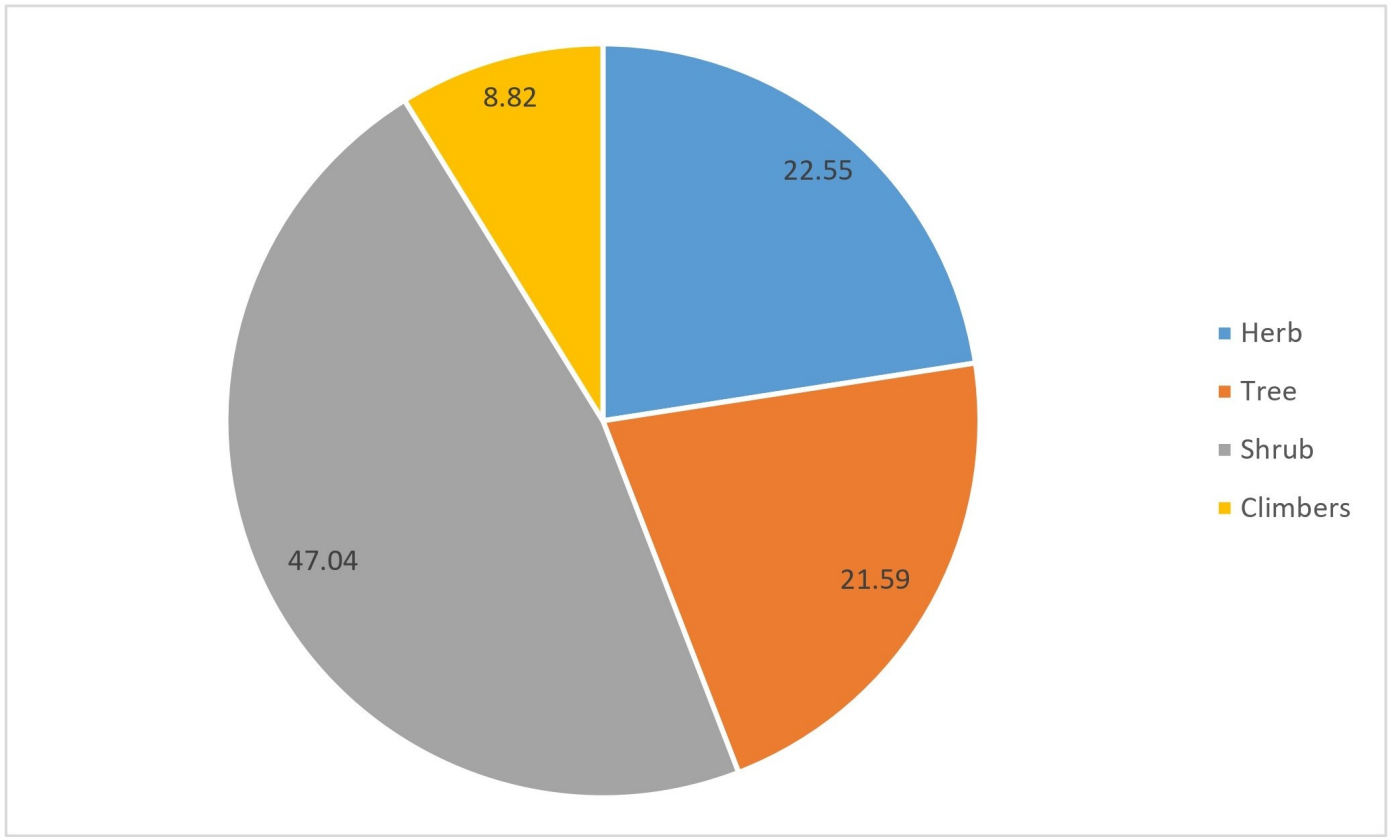
Percentage of plant habit.

Trees (47.04%) were the most commonly used medicinal plants, followed by herbs (22.55%), shrubs (20.59%), and climbers (8.82%), as shown in Fig 4.

### Used plant parts, mode of preparation, and administration

**Fig 5:**
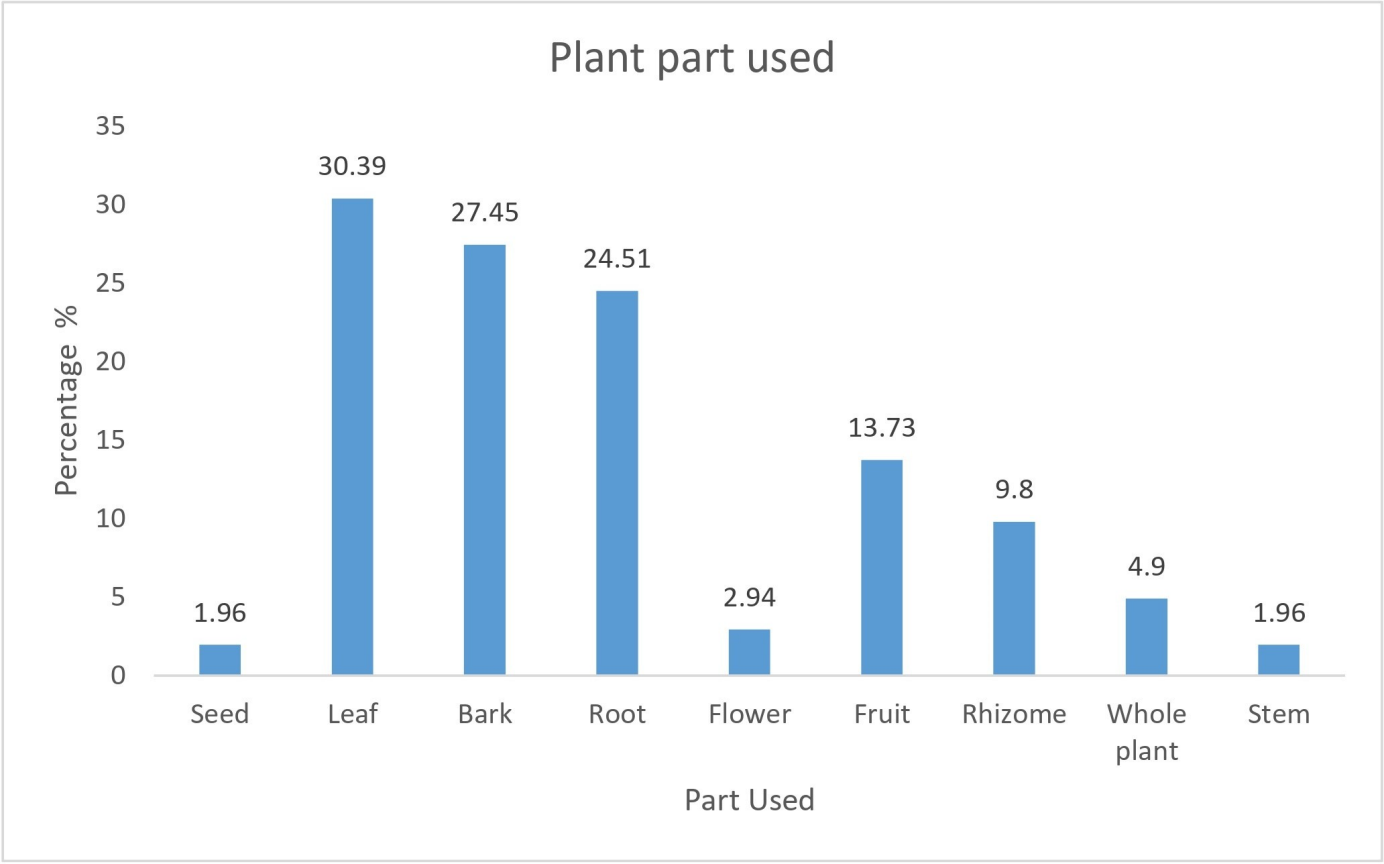
Percentage of plant parts used.

The local people from ethnic groups utilized various plant parts such as leaf, stem, bark, roots, bulbs/rhizome, seeds, fruits, and whole plants. The most common plant parts used were leaves (30.39%), followed by bark (27.45%), and roots (24.51%) (Fig 5).

**Fig 6:**
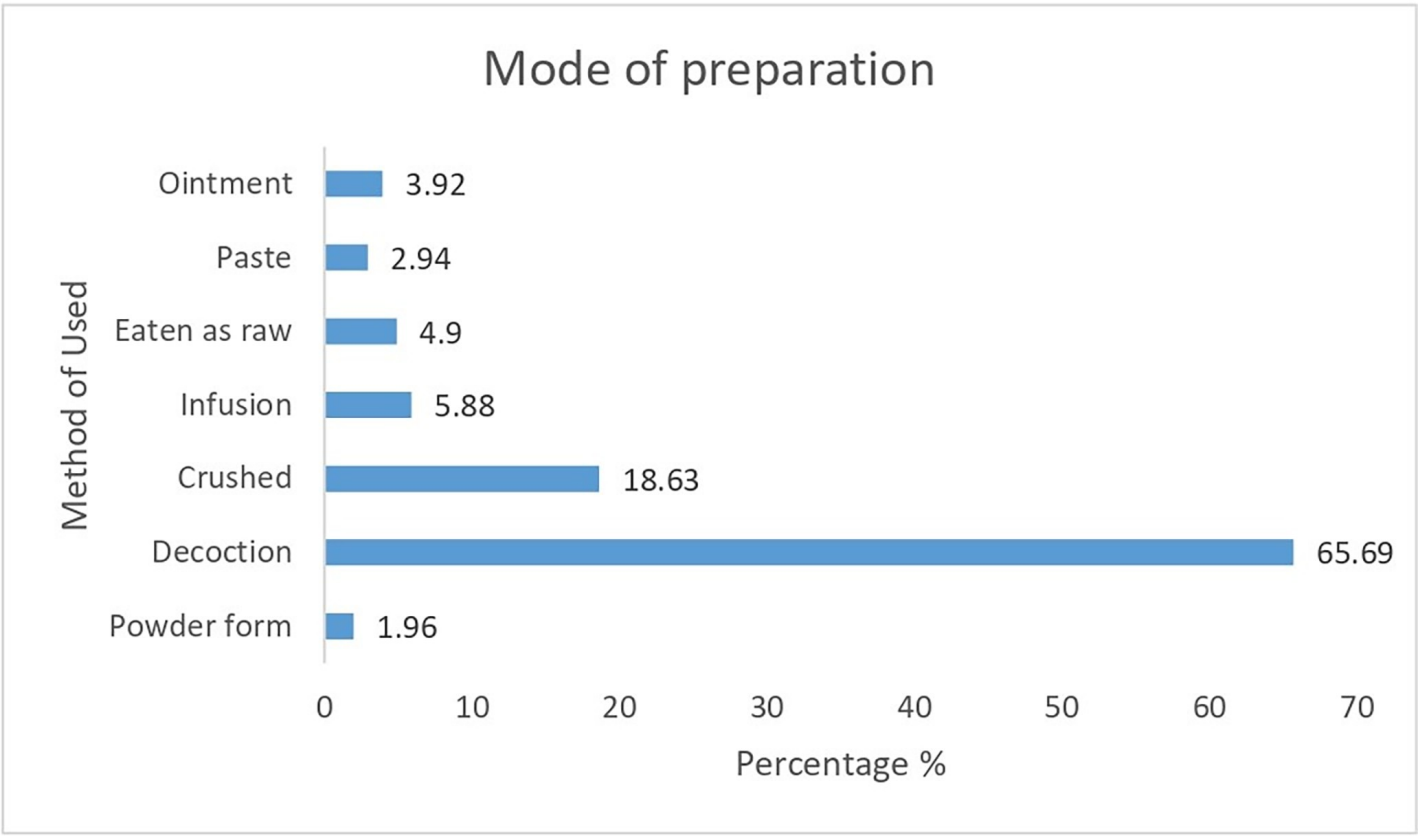
Percentage of the mode of preparation.

Folklore medicines are prepared from fresh or dry plant parts from the study area. The majority of the informants used fresh plants for the preparation of traditional medicines. Decoction (65.69%) was found to be the most common mode of preparation, followed by crushing (18.63%) and infusion (5.88%) (Fig 6).

Herbal medicine can be administered in various ways such as through the skin, inhalation, and inhaling. In the present study, the most common method is oral administration (88 plant species, 86.2%) followed by external application (20 plant species, 19.6%).

### Human ailments treated

In the 17 villages of 5 districts, 102 ethnomedicinally significant plant species utilized to treat a total of 22 human ailments were identified (Table 2). Stomachaches, wounds, and cuts seem to be the most prevalent human ailments among ethnic groups. In these regions herbalists are frequently consulted for various illnesses and the local people would rather consult with traditional healers than modern medicine for various ailments.

The ethnomedicinal plants that were documented for the treatment of various ailments were grouped into 11 different disease categories and calculated for each ailment category. The ICF values ranged from 0.52 to 0.81 (Table 3). The highest ICF was reported for diabetes (0.81), ailment with 38 species, followed by cancer (0.8), liver problems (0.8), and hypertension (0.8).

**Table 3:** Informant consensus factor (ICF) in each ailment category from the study area.

Ailment category	Diseases under category	No of sp	No of use report	ICF value	Most cited taxa with UV	Use of most frequently referenced taxa
**Neoplasms**	Cancer	14	61	0.8	*Phlogacanthus thyrsiformis* (Roxb. ex hardw.) Mabb. UV-1	Cancer
**Metabolic diseases**	Diabetes	19	89	0.8	*Ocimum gratissimum* L. UV-1	Diabetes
**Cardiovascular diseases**	Heart disease, hypertension	14	59	0.8	*Ruehssia macrophylla* (Humb. & Bonpl. Ex Schult.) H. Karst.; UV-0.69	Hypertension
**Respiratory system diseases**	Asthma	4	24	0.5	*Aeschynanthus parviflorus* Spreng.; UV-0.16	Asthma
**Digestive diseases**	diarrhea, dysentery, stomach ulcer, stomachache, constipation	79	167	0.5	*Gelsemium elegans* (Gardner & Champ.) Benth.; UV-0.4	Stomachache
**Skin diseases**	tumor, cuts, wounds, leprosy	9	31	0.7	*Chukrasia tabularis* A. Juss.; UV-0.32	Tumor
**General symptoms**	Haemorrhage, fever and inflammation	11	19	0.4	*Celosia argentea* L.; UV-0.3	Fever
**Genitourinary diseases**	Kidney problem	3	15	0.7	*Hedyotis scandens* Roxb.; UV-0.4	Kidney stone

Where UV represents Use Value.

The fidelity level was calculated for each of the plant species to determine their usefulness for the treatment of various ailments (Table 4).

**Table 4:** Fidelity level of the most commonly used medicinal plants by the informants.

Plant species	Therapeutic uses	Np	N	FL%
*Claoxylon khasianum* Hook. f.	Cancer	52	71	73.2
*Lonicera macrantha* Spreng.	Cancer	41	66	62.1
*Lepionurus sylvestris* Blume	Cancer	52	72	72
*Hellenia speciosa* (J. Koenig) Govaerts	Stomachache	33	47	70.2
*Ocimum gratissimum* L.	Diabetes	45	71	63
*Zanonia indica* L.	Stomach ulcer	38	69	55
*Curcuma caesia* Roxb.	General diseases	19	31	61
*Aeschynanthus parviflorus* Spreng.	Heart disease	9	20	45
*Mucuna pruriens* (L.) DC.	Liver problem	12	21	57
*Lobelia angulata* G. Forst	Hypertension	21	45	47
*Cirsium shansiense* Petr.	Skin Problem	18	32	56.3
*Polygonatum cirrhifolium* (Wall.) Royle	Respiratory problem	14	30	47
*Hedyotis scandens* Roxb.	Kidney problem	28	48	58.3
*Plantago major* L.	Diabetes	51	82	62
*Helicia excelsa* Blume	Stomachache	23	37	62.2

Where Np = number of informants who claimed the use of plant species to treat a particular illness; N = number of informants who use the plant as ethnomedicine to treat a given disease; FL% = percentage of fidelity level.

The plant use value (UV) was evaluated from the documented medicinal plants, and the value ranged from 0.14 to 1. *Claoxylon khasianum* Hook.f., *Lepionurus*. *sylvestris* Blume, *Ocimum gratissimum* L., and *Phlogacanthus thyrsiformis* (Roxb. ex Hardw.) Mabb. showed the highest (1) use value (Table 2).

The cultural value was calculated among the documented medicinal plants utilized by the various ethnic groups from the study area. Plant species with high cultural values are shown in Table 5. The high value of CV exhibit strong agreement with the survey culture and for determining the people’s common knowledge.

**Table 5:** High cultural values (CV) of medicinal plants from the study sites.

Species name	CV
*Phlogacanthus thyrsiformis* (Roxb. ex Hardw.) Mabb.	31
*Ocimum gratissimum* L.	32
*Lepionurus sylvestris* Blume	24
*Leea compactiflora* Kurz	23
*Ruehssia macrophylla* (Humb. & Bonpl. ex Schult.) H. Karst.	21.4
*Eurya acuminata* DC.	14.5
*Amomum maximum* Roxb.	10.8
*Lonicera macrantha* Spreng.	4.6
*Lobelia angulata* G.Frost.	4
*Zanonia indica* L.	6.7

### Threats to medicinal plant knowledge and use

According to the informants, agricultural growth (47%) posed the biggest threat to medicinal plant resources followed by population expansion (32%), deforestation (29), overgrazing (6%), and forest fire (2.5%). Various conservation methods were suggested by the informants. As a result, 39% of the informants opined that plantation was the best way to preserve and safeguard herbal medicines followed by an awareness campaign (19%) and soil and water conservation (15%).

## Discussion

Local traditional medicinal knowledge is typically passed down from an older practitioner to a male successor rather than a female successor. The fact that traditional healers typically prefer to impart their knowledge of local medicinal plants to other men might account for the study area’s high proportion of male informants. Similar finding on the predominance of men was also found in Mizoram and other countries [4,14,24–26].

Although the present study highlighted the ethnomedicinal plants with special emphasis on cancer and other cardiovascular diseases, 102 plants have been documented for medicinal purposes. Compared to previous ethnobotanical studies from Mizoram, the number of medicinal plants documented in the present study was higher such as the Champhai district [24], the Western region of Mizoram [14]. This indicates that the study area has diverse flora and rich traditional knowledge used in the management of various ailments. The utilization of medicinal plants as their primary healthcare source from the wild has been reported from the western region of Mizoram [14].

A greater capacity for adaptability of the species in the family across a larger range of elevations may explain the highest number of medicinal plants from Fabaceae (n = 8) that has been observed. Studies conducted in Mizoram, as well as other regions [4,25,26], also revealed a substantially higher number of Fabaceae plants that are used as remedies.

The local people among various ethnic groups mainly rely on natural vegetation for medicinal plants, revealing that the practice of planting or cultivating medicinal plants is lacking. Therefore, overexploitation of natural vegetation may pose a serious threat in the study areas. Previous findings also showed that herbal medicines were mostly collected from natural vegetation [14,24].

Trees were the most prevalent plant life forms used for therapeutic purposes. However, previous studies from other regions reported the dominance of herbs, and shrubs [14,24]. Trees are the most common habit in the study areas for medicinal purposes, so the collection of the plants may pose a threat to conservation. However, since leaves are primarily harvested for medical purposes, harvesting leaves has little effect on the survival of plant species, while harvesting the entire plant or its roots may have a negative impact. The local practitioners gathered the plant materials from the forest due to the lack of medicinal plant gardens.

Leaves are the most commonly reported plant components used in traditional medicine preparations in India [11] and other countries [27]. Compared to other plant parts, leaves can be easily obtained in large quantities, which may account for their higher utilization rates. Additionally, it is the site of photosynthesis as well as components of phytochemicals that are pharmacologically used for the treatment of various diseases [28].

The widespread use of fresh plants in traditional medicines may be related to the increased perceived efficacy of fresh plant parts that may be lost after drying. As a result of the evaporation and degradation of the bioactive compounds during drying, dry forms of plant-based remediation have low efficacy. The freshly collected medicinal plants were preferred in previous studies done in India [14,24] and other countries [4,25,26].

Decoctions (hot aqueous extract/boiling) were mentioned as the most frequently used method of preparing herbal medicines. When compared to cold extraction, boiling is more effective at extracting plant materials and preserving herbal remedies for a longer time. Previous studies have also found decoction as the most popular method of preparation [24]. The method of decoction was similar for all the ethnic groups under investigation.

Herbal medications are typically taken orally, and various herbal remedies frequently employ this mode of administration [29,30]. The popularity of oral use was attributed to its ease of administration and the present finding is comparable with previous studies [24,25]. Traditional healers in the area frequently use different additives (honey, sugar, etc.) to improve the flavour and taste of some oral medications. *Z*. *indica* and *R*. *serpentina* have been used to mix with honey, while *D*. *indica* has been mixed with salt to improve the taste. Additives are essential for decreasing discomfort, improving flavor, and minimizing negative effects such as vomiting [31].

The average number of medicinal plants cited by male and female informants in the study area did not show a significant difference that demonstrates knowledge is equitably distributed among all family members. Both males and females are in charge of providing basic healthcare for their families. A study by Tahir et al. [25] also found the similar result.

There was a significant difference between the key informants and the general informants as the previous were more knowledgeable than the latter which was in comparable with the previous studies [32]. Their extensive training and strict confidentiality when using medicinal plants, may be explained by their many years of expertise [25]. The younger people’s knowledge of traditional medicinal plants was low compared to that of older people as evidenced by the significant difference between age groups, which was similar to previous studies [24]. Older people have greater knowledge of the ethnomedicinal uses of plants than younger people. This reduced degree of knowledge may be explained by elements like modern education and oral transmission [26]. Younger people, on the other hand, generally show little interest in traditional medicines, and if nothing is done to encourage them, there seems to be a risk of loss of traditional knowledge. There was no significant difference in the mean number of medicinal plants reported among educational levels, similar to previous work [32]. However, the results showed that low-education-level informants were more knowledgeable than higher-education informants. This may be related to the detrimental effects of contemporary education on understanding traditional medicine [25].

Traditional remedies are still favoured in primary healthcare in Mizoram [24] and other countries [33]. Most of the study areas are still underdeveloped and remote places, lack conventional healthcare facilities, and experience widespread poverty. Traditional medicines used in the study area communities meet their diverse healthcare needs.

Among ethnic groups, medicinal plants are used to treat various ailments from minor to chronic illnesses. However, in Mizoram, illnesses such as cancer and cardiovascular diseases are very common; therefore, the present investigation mainly focused on the above-mentioned diseases. In our study, the analysis showed that most of the ailment categories had high ICF values. When the plants have high diversity, their ICF value is also high. If the plant diversity is low, the ICF value also decreases [21].

Among the documented medicinal plants, *C*. *khasianum* had the highest fidelity level (FL) value (73.2%), followed by *L*. *sylvestris* (72%) and *H*. *speciosa* (70.2%) for cancer disease. In addition to cancer treatment, *C*. *khasianum* has also been used for the treatment of tumours [11]. *L*. *sylvestris* has also been used to treat diabetes and stomachache [14]. The level of a species’ importance to a specific disease can be determined using FL, which depicts the proportion of survey participants who mentions using a particular plant species for the same primary objective [34]. Several studies have opined that medicinal plants with high FL should be taken into consideration, as they are popularly used as potential candidates for further phytopharmacological research [35,36].

The *O*. *gratissimum* had the highest cultural value, followed by *L*. *sylvestris*, and had a wide range of medicinal value, since the medicinal plant has been used for various illnesses, particularly in the southern region of Mizoram. This could be the cause of the high level of cultural value among the informants because cardiovascular diseases such as diabetes and hypertension are prevalent in these areas.

The rate of agreement and sharing of knowledge about and use of medicinal plants among informants is more likely to be higher when the UV value is higher [37]. *C*. *khasianum*, *L*. *sylvestries*, *O*. *gratissimum*, and *P*. *thyrsiformis* were the species that were used the most frequently with a use value of 1 and credited for their use in the treatment of various ailments, and all the informants were aware of their effectiveness. The quantitative analysis showed that these species were the most pertinent species with high use value, cultural value, ICF, and FL. A plant with a higher fidelity level may also be more effective to treat a particular disease. The values of fidelity level can be used as a guide to finding medicinal plants with more healing potential and those that need to be further studied in terms of phytochemical composition and identification of bioactive compounds. According to Chaudhary et al. [38], plants with low usage value were at risk of being misremembered and passed on to the next generation, which might eventually perish. On the other hand, the use of understanding a plant’s used value was for the ease of pharmacological study and the dependability of their application. The cultural importance index (CI) explains both the value of each species as well as the distribution of its uses. It is safe to infer that the CI index is a useful instrument for emphasizing those species that have a high level of agreement with the survey culture and for identifying the shared knowledge of the people. The present study showed that indigenous informants’ knowledge of ethnomedicine practices were highly consistent and that they used the same plants to treat various ailments. The use of quantitative indices was crucial for identifying the most valuable plants, understanding their cultural significance, and creating conservation plans. Various phytochemical compounds are known to exist in plants with high use value and cultural value. The quantitative indices demonstrated a significant value, suggesting that they can serve as a reliable source for future ethnopharmacological research.

Due to habitat destruction and overharvesting of species known to be medicinal, the risk to medicinal plants is rising [39]. In the study area, the local people rely on medicinal plants not only for medicinal purposes but also for food, construction, firewood, etc. The present study shows that traditional therapeutic knowledge also becomes threatened due to a lack of proper documentation and a lack of interest among the younger generation. Therefore, it is crucial to implement policies to enhance the preservation, development, and sustainable use of medicinal plants and their traditional knowledge among the local people. Further studies and documentation of traditional therapeutic knowledge are essential at this juncture. Also to improve the effectiveness of the traditional medicines used by the local people, advanced technology and pharmacology methods such as innovative extraction technologies such as semi-bionic extraction, microwave-assisted, ultrasonic-assisted, and enzyme-assisted extraction, and sophisticated new methodologies and instrumentation such as HPLC-MS, LC-MS, GC-MS have made it possible to re-evaluate the body of traditional knowledge, determine the chemical components of plant extracts, identify active compounds and develop novel drugs.

The southern region of Mizoram is inhabited by various ethnic groups, such as Lushai, Lai, Mara, Bawm, Chakma, and Bru. The ethnic groups usually speak their local dialects and retain their respective cultures, customs, and festivals. People who live in these areas mainly use herbal medicine for the treatment of various ailments. Due to the availability, acceptability, and affordability of traditional medicines, the local inhabitants mainly relied on traditional healthcare systems. In the recent past, there has been a major global advancement in modern healthcare. Technology development and scientific advancement led to the development of novel and creative treatments for the ailment. Life expectancy has grown and mortality has reduced as a result of advances in medical research. Even with this enormous and amazing advancement, accessibility is still a major concern for many people worldwide. Most developing and impoverished nations lack access to contemporary medical treatment. Regarding the indigenous people of the study areas, it is accurate. Due to their remote and inaccessible living conditions, local people find it extremely difficult to receive healthcare treatments. As a result, many of them have long only used traditional medicine. The inhabitants in the study area still live a simple life, and most of their houses are built with bamboo and wood. The economic activities of the ethnic groups are mainly agriculture, selling of cash crops, and animal husbandry.

### EthnobotanyR chord plot

**Fig 7:**
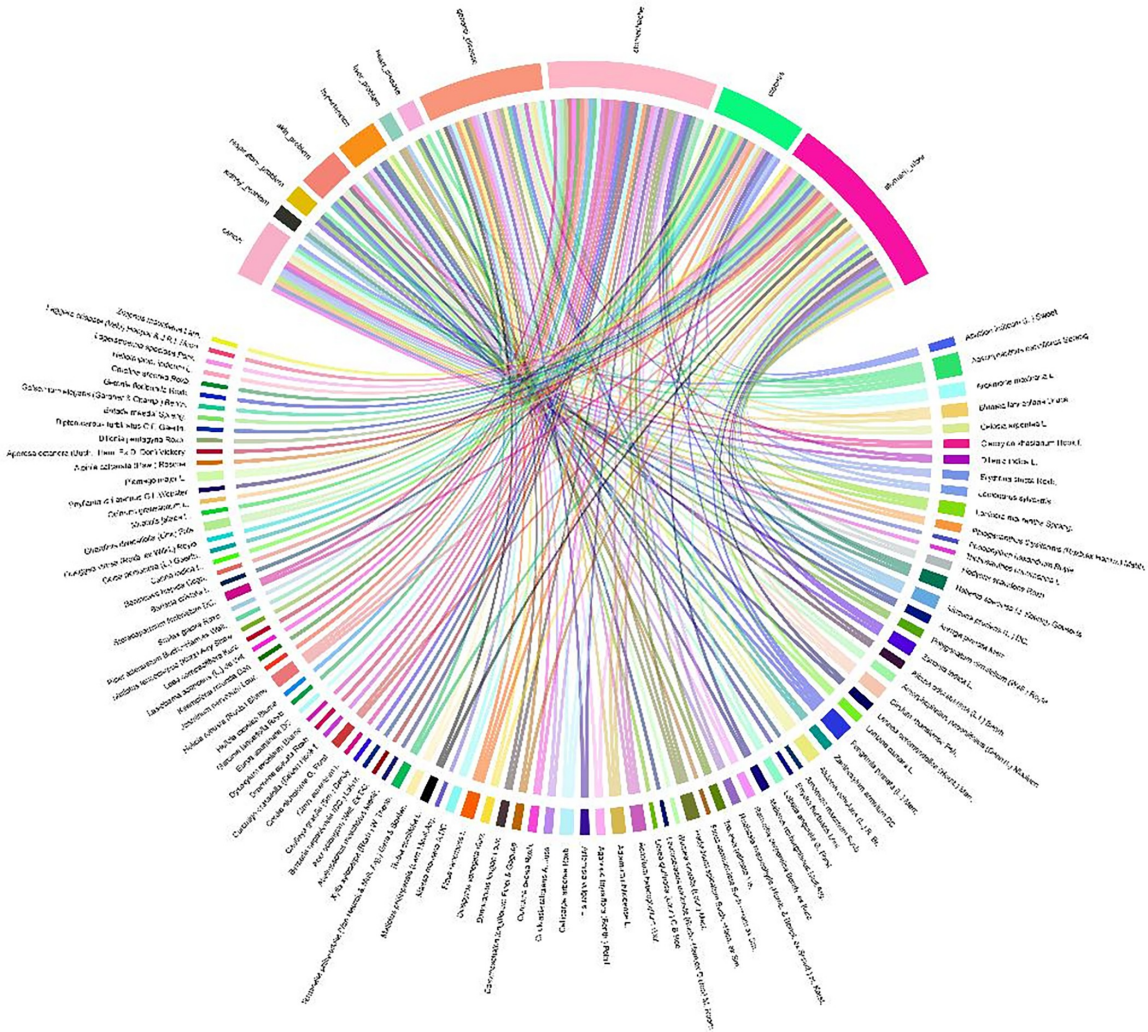
Chord plot using EthnobotanyR showing various medicinal plants used for the treatment of various ailments.

The chord diagram in Fig 7 highlights the varied applications of the studied plant species. It was discovered that 14 species were found useful for the cancer treatment, 3 for kidney problems, 4 for respiratory problems, 9 for skin problems, 10 for hypertension, 4 for heart diseases, 37 for stomach problems, 19 for diabetes, 39 for stomach ulcers, and 27 for general disease. A large number of stomach-related species might be attributed to the prevalence of stomach-related sickness in Mizoram [40]. It was also found that certain species might have multifunctional purposes in ethnobotany, such as *A*. *heterophyllum*, which was used for both general sickness and cancer, and *T*. *palmata*, which was used for hypertension was also used for stomachache. The analysis also showed that the ethnobotanical usage of a particular plant in terms of disease control was varied, highlighting the importance of the plants under current investigation.

### Cross-cultural analysis

**Fig 8:**
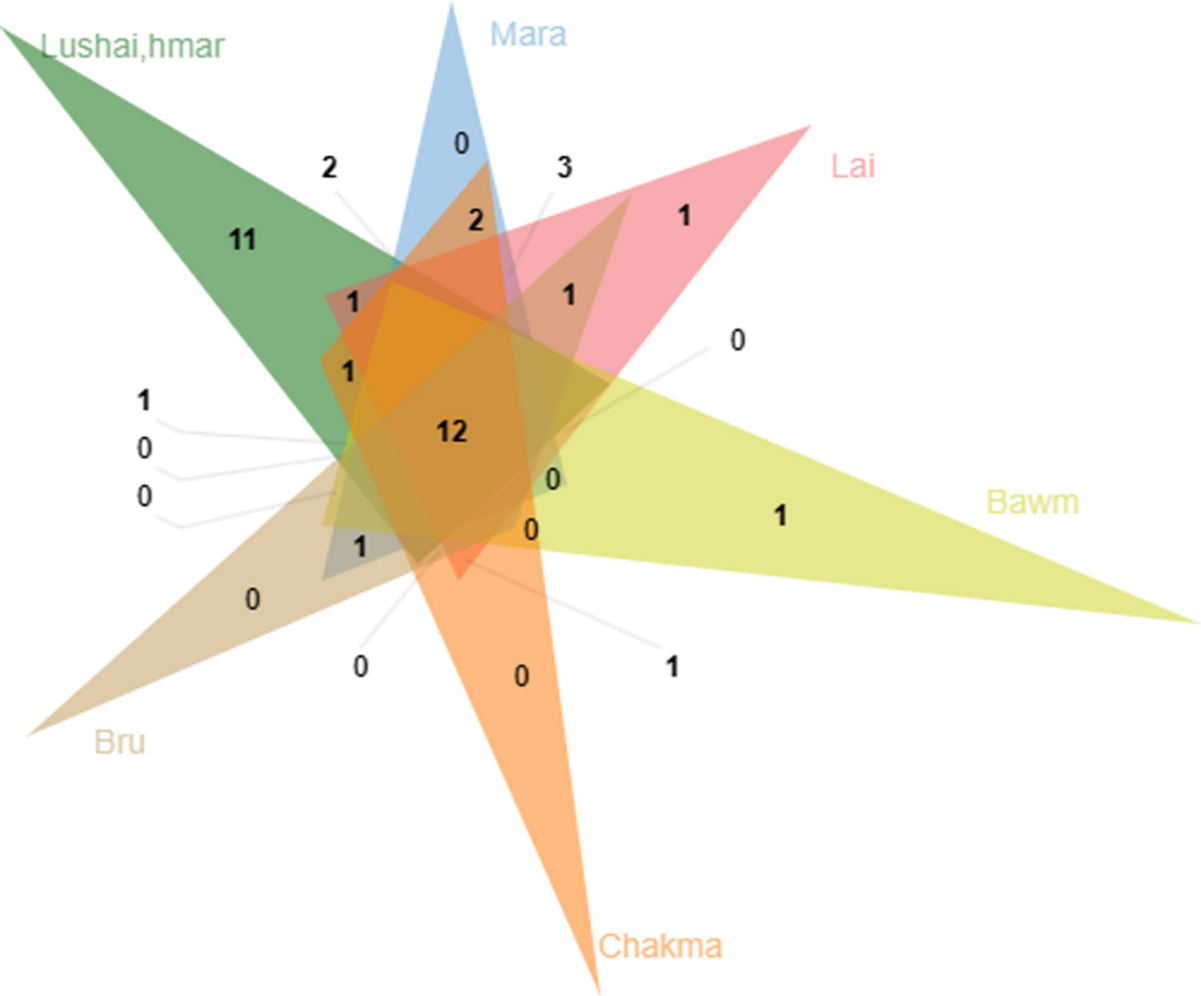
Venn diagram illustrating the similarity of medicinal plant uses among various ethnic groups in Mizoram, India.

The cross-cultural analysis was performed among the studied ethnic groups. The Venn diagram (Fig 8) showed that the maximum number of plant use among the ethnic groups was reported by the Lushai group, while the Bru group showed a minimum number of use of medicinal plants. The Lushai and Mara ethnic groups showed higher similarity whereas the least similarity was found between the Bru and Lai groups. A total of 12 plants were found to be widely used by all ethnic groups in a cross-cultural study of plant resources. The medicinal plants which were commonly used among the ethnic groups had high medicinal value.

### Health significance of the present study for the community

Medicinal plants have played an important role in human health and have the potential to treat various illnesses such as cancer, ulcers, tumors, diarrhea, etc. The utilization of natural products for medicinal purposes has been studied and documented in various countries, however, there is an urgent need to conduct the medicinal potential on a scientific foundation. Several species have been related to cure stomachache, indigestion, diarrhea, and ulcers, as well as therapeutic effects against various ailments. These plant species such as *C*. *indica*, *C*. *caesia*, *E*. *rheedii*, *T*. *palmata*, *A*. *vulgaris*, *C*. *arborea*, *H*. *spicatum*, *Z*. *mauritiana*, and *P*. *major* have been reported for the treatment of various ailments in Bangladesh, Pakistan, Nepal, and Afghanistan [41–44]. It has also been reported that the plants *A*. *scholaris* and *R*. *serpentina* have been used for the treatment of dysentery and hypertension in Bangladesh similar to the present study [45]. It is necessary to screen the potential anti-stomachache, anti-diarrheal, and anti-ulcer agents to isolate novel bioactive compounds for new drug formulations. Additionally, medicinal plants have drawn a lot of attention lately. Some local healers have prepared these plants—the juice of *C*. *longa*, for instance, is said to have anticancer and stomach ulcer-healing properties—and are selling them in the marketplace for between 150 and 200 Indian rupees per liter. However, the majority of people living in villages rely mostly on agricultural products. Cutting down trees and lowering forest timber and timber capacity are two main factors for the inability to sustain local industry. An approach like this aligns with the documentation of ethnobotanical knowledge in the relevant field. Documenting the ethnobotanical data is crucial to preserve it before it is lost.

### Novelty of the present study and future prospects

The current study locations encompass a range of ethnic groups found in Mizoram, and the research area is perfect for a variety of flora because it includes two forest reserves: Lengteng Wildlife Sanctuary and Dampa Tiger Reserve. Most of the vegetation was specific to each region. The southern region of the study areas inhabited by various ethnic groups such as Bawm, Chakma, Mara, and Lai was dominated by *O*. *gratissimum*, *H*. *scandens*, and *P*. *major* while the Northern region was inhabited by Lushai, Hmar, Bru, dominated by *L*. *sylvestris*, *L*. *angulata*, *F*. *virosa*, *A*. *octandra*, *Z*. *mauritiana*, *T*. *palmata*, *R*. *macrophylla*, and *C*. *arborea*. Previous researchers have not yet reported on the economic relevance of therapeutic plants in the study area. Nonetheless, the current investigation also concentrated on the financial importance of the identified medicinal plants found in the research region. Accordingly, the study demonstrated the economic importance of specific plants, like *Z*. *mauritiana*, *T*. *palmata*, and *R*. *macrophylla*, which are grown by indigenous people on their farms and sold to meet their basic requirements. These plants have a significant function in the marketplace.

## Conclusions

Mizoram is home to a diverse range of plant species that are used as remedies for a variety of ailments. Natural products are still popular in remote areas where access to modern healthcare facilities is difficult. Locals still use herbal remedies to treat a variety of illnesses as part of their daily lives and cultures, which calls for the preservation of the forest’s integrity and its indigenous users’ herbal medicine knowledge. A total of 102 ethnomedicinal plant species utilized by seven ethnic groups present in Mizoram to treat various human ailments were investigated and documented. This result illustrates the rich diversity of medicinal plants in Mizoram. These medicinal plants play an essential role in the indigenous people’s healthcare system. In a cross-cultural assessment of plant resources, 12 plants were discovered to be widely used by all ethnic groups. The medicinal plants that were widely used by ethnic groups had great medical value. The present study showed that the documented plants have the potential for the discovery of new drugs and nutraceutical formulations. Traditional medical knowledge and medicinal plants, on the other hand, are gravely threatened by rapid economic development for a variety of reasons. As a result, policies and practices for the protection of medicinal plants and the traditional knowledge linked with them are required.
